# *In Vivo* Sub-chronic Treatment with Dichlorvos in Young Rats Promotes Synaptic Plasticity and Learning by a Mechanism that Involves Acylpeptide Hydrolase Instead of Acetylcholinesterase Inhibition. Correlation with Endogenous β-Amyloid Levels

**DOI:** 10.3389/fphar.2017.00483

**Published:** 2017-07-25

**Authors:** Gonzalo García-Rojo, Fernando Gámiz, Estíbaliz Ampuero, Daniel Rojas-Espina, Rodrigo Sandoval, Carlos Rozas, Bernardo Morales, Ursula Wyneken, Floria Pancetti

**Affiliations:** ^1^Laboratory of Environmental Neurotoxicology, Department of Biomedical Sciences, Faculty of Medicine, Universidad Católica del Norte Coquimbo, Chile; ^2^Laboratory of Neuroscience, Faculty of Medicine, Universidad de Los Andes Santiago, Chile; ^3^Laboratory of Neuroscience, Department of Biology, Faculty of Chemistry and Biology, Universidad de Santiago de Chile Santiago, Chile

**Keywords:** acylpeptide hydrolase, acetylcholinesterase, dichlorvos, hippocampus, synaptic plasticity, learning

## Abstract

Acylpeptide hydrolase (APEH) is a serine hydrolase that displays two catalytic activities, acting both as an exopeptidase toward short *N*-acylated peptides and as an endopeptidase toward oxidized peptides or proteins. It has been demonstrated that this enzyme can degrade monomers, dimers, and trimers of the Aβ_1-40_ peptide in the conditioned media of neuroblastoma cells. In a previous report, we showed that the specific inhibition of this enzyme by the organophosphate molecule dichlorvos (DDVP) triggers an enhancement of long-term potentiation in rat hippocampal slices. In this study, we demonstrate that the same effect can be accomplished *in vivo* by sub-chronic treatment of young rats with a low dose of DDVP (0.1 mg/kg). Besides exhibiting a significant enhancement of LTP, the treated animals also showed improvements in parameters of spatial learning and memory. Interestingly, higher doses of DDVP such as 2 mg/kg did not prove to be beneficial for synaptic plasticity or behavior. Due to the fact that at 2 mg/kg we observed inhibition of both APEH and acetylcholinesterase, we interpret that in order to achieve positive effects on the measured parameters only APEH inhibition should be obtained. The treatment with both DDVP doses produced an increase in the endogenous concentration of Aβ_1-40_, although this was statistically significant only at the dose of 0.1 mg/kg. We propose that APEH represents an interesting pharmacological target for cognitive enhancement, acting through the modulation of the endogenous concentration of Aβ_1-40_.

## Introduction

Acylpeptide hydrolase (APEH) is a homomeric tetramer that belongs to the prolyl oligopeptidase family of serine hydrolases (Rosenblum and Kozarich, 2003) and catalyzes the hydrolysis of several peptides that possess an acylated *N*-terminal amino acid to generate an acylated amino acid and a free *N*-terminal peptide (Scaloni et al., 1992; Polgar, 2002). In mammals, APEH acts in coordination with the proteasome to clear cytotoxic denatured proteins from cells (Fujino et al., 2000b; Shimizu et al., 2004; Palmieri et al., 2011) and it has also been described that a truncated form of the enzyme displays endopeptidase activity in bovine and human lenses (Senthilkumar et al., 2001; Santhoshkumar et al., 2014). At present, there is little information about the role of APEH in the nervous system. Four reports, two from our group and the other two from the group of Carmela Abraham at Boston University, have been published with findings that have contributed to the understanding of the role of this enzyme in neuronal tissue. Taken together, these reports demonstrate the involvement of APEH activity in the modulation of synaptic activity (Olmos et al., 2009), its localization in synapses of the rat telencephalon (Sandoval et al., 2012), and the ability of the enzyme found in the conditioned medium of neuroblastoma cells to degrade monomers, dimers, and trimers of the Aβ_1-40_ peptide (Yamin et al., 2007, 2009).

The catalytic activity of APEH can be pharmacologically inhibited by the organophosphate compound dichlorvos (2,2-dichlorovinyl dimethyl phosphate, DDVP) (Quistad et al., 2005). Interestingly, DDVP is the active metabolite of metrifonate, a drug used some years ago as a cognitive enhancer for the treatment of patients with dementia, particularly of the Alzheimer’s type (Hinz et al., 1996; Cummings et al., 2001). This compound was originally considered as an inhibitor of acetylcholinesterase (AChE), until it was demonstrated to preferentially inhibit APEH activity rather than AChE as its primary target (Richards et al., 2000). This evidence led us to hypothesize that APEH could play a role in the modulation of synaptic plasticity processes that underlie cognitive abilities (Silva, 2003; West and Greenberg, 2011). To test this hypothesis, we performed long-term potentiation (LTP) experiments in rat hippocampal slices exposed to DDVP *in vitro*. We used LTP as an experimental approach to determine the effects of DDVP on synaptic plasticity, which is considered the cellular mechanism of learning and memory (Bliss and Collingridge, 1993; Cooke and Bliss, 2006). Briefly, we observed that the inhibition of APEH by the acute exposure of rat hippocampal slices to DDVP induced a significant increase of LTP at the glutamatergic synapses of rat hippocampal slices *in vitro*. This effect was only seen at a specific window of dosage and exposure time. We also showed that the effect occurred through an indirect mechanism involving the activation of post-synaptic α7 nicotinic receptors and could be mediated by the endogenous peptide substrate of APEH (Olmos et al., 2009).

In this work, we have addressed the question of whether the improvements observed in synaptic plasticity triggered by acute inhibition of APEH *in vitro* can be replicated *in vivo*. For this, we administered sub-chronic treatments of DDVP to young male rats at a range of doses, to determine which doses produced a significant enhancement of LTP and a specific APEH inhibition. We also investigated if this effect was correlated with behavioral improvements in learning and memory and with changes in the endogenous Aβ levels.

## Materials and Methods

### Animals

Three-week-old male Sprague Dawley rats (post-natal day 21) were housed two per cage with food and water available *ad libitum* under a 12:12 h light–dark cycle. The room temperature was maintained at 22–23°C. Rats were subcutaneously injected with various doses of DDVP (liquid, analytical purity, Dr. Ehrenstorfer Lab, Augsburg, Germany) dissolved in corn oil as the vehicle. Control rats were injected with vehicle alone. The doses studied were 0.03, 0.1, 0.5, 2, and 6 mg/kg. The injections were administered daily for 28 days. At day 29, rats used for electrophysiological recordings were anesthetized with halothane gas and decapitated, and their brains were rapidly removed for hippocampal slice preparation. Another group of 36 rats were treated for 28 days with either a low or a high dose of DDVP and used for the behavioral evaluation (control, *n* = 18; 0.1 mg/kg DDVP, *n* = 8; 2 mg/kg DDVP, *n* = 10). All of the protocols dealing with the maintenance and handling of animals were followed as stated in the Bioethical Guidelines of the Universidad Católica del Norte and in the National Research Commission guidelines.

### Hippocampal Slice Preparation

After decapitation of the rats, the brains were rapidly immersed in ice-cold dissection buffer containing 212.7 mM sucrose, 5 mM KCl, 1.25 mM NaH_2_PO_4_, 3 mM MgSO_4_, 1 mM CaCl_2_, 26 mM NaHCO_3_, and 10 mM glucose at pH 7.4. The hippocampus was dissected and transverse slices (400 μm thick) were obtained from the middle third portion using a vibratome (Model HA752, Campden Instruments, Leicester, United Kingdom). The slices were transferred to an interface storage chamber containing artificial cerebrospinal fluid (ACSF) saturated with 95% O_2_/5% CO_2_ and were kept at least 1 h at 37°C prior to recording. The ACSF contained 124 mM NaCl, 5 mM KCl, 1.25 mM NaH_2_PO_4_, 1.0 mM MgCl_2_, 2.0 mM CaCl_2_, 26 mM NaHCO_3_, and 10 mM glucose at pH 7.4. Single slices were then transferred to a recording chamber where they were kept completely submerged in ACSF and continually perfused (2 mL/min).

### Extracellular Field Recording and LTP Induction

Field responses were evoked by electrical stimulation delivered every 15 s to the Schaffer collateral pathway using bipolar electrodes and recorded in the *stratum radiatum*, for measuring field excitatory post-synaptic potentials (fEPSP) slopes, or the *stratum pyramidale*, for measuring population spike (PS) amplitudes of the CA1 hippocampal area. The recording electrodes were glass micropipettes (1–3 MΩ) filled with ACSF. At the beginning of each experiment, stimulus/response curves were generated by increasing the intensity of the stimulus to elicit 50% of the maximum response. LTP was elicited after 10–15 min of a stable baseline by theta burst stimulation (TBS) consisting of five trains of stimulus with an inter-train interval of 10 s. Each train consisted of 10 bursts at 5 Hz, each burst having four pulses at 100 Hz. After TBS, data acquisition lasted for 1 h. Data were acquired using an extracellular amplifier (DAGAN) and a data acquisition board (National Instruments, United States) controlled through IGOR software (Wavemetrics Inc., United States).

### Input/Output Curves

Input/output ratios were measured in response to single electrical stimulation for both the control and DDVP-treated rats. The fEPSP responses elicited by stimulus strength intervals of 20 μA were measured at the CA1 *stratum radiatum* area. When the stimulus strength reached 100 μA, increments were added in steps of 50 μA up to a maximum of 500 μA. Three responses were collected and averaged for each stimulus increment. To construct the input/output curves, data were averaged across all slices measured for each group (control and DDVP-treated).

### Paired-Pulse Stimulation

Paired-pulse facilitation (PPF) experiments were performed in the *stratum radiatum* of the CA1 hippocampal area. Biphasic, constant current, 100 μs stimuli were delivered in pairs at 15 s intervals. The paired pulses were administered with doubling of the interstimulus interval, starting at 20 ms and ending 2560 ms apart. The stimulus intensity was adjusted in such a way that the field response for the first pulse (P1) was 50% of the maximum amplitude. Paired-pulse inhibition (PPI) was performed in the *stratum pyramidale* of CA1. Stimuli were delivered in pairs at 15 s intervals. Paired pulses were given 13 ms apart.

### Preparation of Tissue Extracts and Protein Determination

After each electrophysiological experiment, the slice was recovered from the perfusion chamber and frozen at -80°C until needed, at which point the slices were homogenized at 4°C in a buffer containing 50 mM Tris-Cl, 1 M NaCl, 50 mM MgCl_2_, and 1% Triton X-100 at pH 7.4. Protein concentrations were determined using the Bradford method with bovine serum albumin as the standard (Bradford, 1976).

### Acetylcholinesterase Activity Assays

The AChE activities of the brain slice homogenates were determined spectrophotometrically by monitoring the hydrolysis of *S*-acetylthiocholine iodide at 30°C (ε_406_ = 13300 M^-1^ cm^-1^) according to Ellman’s method (Ellman et al., 1961). Briefly, acetylthiocholine iodide was used as a synthetic substrate for AChE. The sample (7 μL containing 30–50 μg of protein) was mixed with 1 mL of 0.25 mM dithiobisnitrobenzoate (DTNB) in 50 mM phosphate buffer at pH 7.9 and the resulting mixture was incubated at 30°C in a water-jacketed cuvette holder. The reaction was initiated by adding 30 μL of the substrate (5 μmol). The final volume of the reaction mixture was 1037 μL. The thiocholine released by the substrate hydrolysis reacts with DTNB to afford 5-thio-2-nitrobenzoate, which was quantified at 406 nm using a Specord 205 spectrophotometer (Analytik Jena, Germany). The enzymatic activity was normalized in function of the protein content in the assay.

### Acylpeptide Hydrolase Activity Assays

The measurement of APEH activity was performed as described previously (Perrier et al., 2002). Briefly, APEH activity was assayed by monitoring the hydrolysis of the synthetic substrate N-acetyl-L-alanine p-nitroanilide (AANA). The samples (10 μL containing 100–200 μg of protein) were mixed with 50 mM phosphate buffer at pH 8.0 in a total volume of 1 mL and incubated at 37°C in a water-jacketed cuvette holder. The reaction was initiated by adding 10 μL of substrate (1 nmol) dissolved in dimethyl sulfoxide. The final volume of the reaction mixture was 1020 μL. The p-nitroaniline released was determined quantitatively using a Specord 205 spectrophotometer (Analytik Jena, Germany) by measuring the absorbance at 410 nm using ε_410_ = 8800 M^-1^ cm^-1^. The enzymatic activity was normalized in function of the protein content in the assay.

### Morris Water Maze Behavioral Test

Spatial learning and memory was measured using the Morris water maze test (Morris, 1984). The apparatus consisted of a circular black water tank (180 cm diameter, 50 cm depth) located in a room with dim indirect light. The pool was filled with 30 cm of water heated to 22°C. A circular black escape platform (11.5 cm diameter) was used. The walls of the procedure room contained visual cues for spatial orientation. The experiment was performed in two different phases. In the first phase, we compared rats injected with 0.1 mg/kg of DDVP (*n* = 8) and the control injected with only the vehicle (*n* = 8). In the second experiment, we compared rats injected with a DDVP dose of 2 mg/kg (*n* = 10) with the control (*n* = 9). The rats were moved to the procedure room 15 min prior to training. Each rat took part in four trials each day with 1 min inter-trial intervals. On the 1st day of training, a visual cue task was conducted. In this phase, the platform was above water level and a small flag was present to facilitate its localization. In each of the four trials, the platform was moved to one of the different quadrants and the rats were released from the opposite quadrant facing the pool wall. Each trial lasted 90 s or until the animal reached the platform. In the case that the time expired, the rats were gently directed to the escape platform and kept there for 15 s. This visual cue task was conducted to detect motor, motivational, or visual problems and differences between the groups. The spatial learning task began on the 2nd day. In this phase, the platform was hidden 2 cm below the water surface and located in the center of the southwest quadrant. In each trial, the rat was placed into the pool at one of the four cardinal compass points. The order of these start locations was pseudorandomly varied throughout the training. If the rat failed to find the escape platform within the allowed time, it was placed onto the platform and a latency of 90 s was assigned. The rats were trained in this phase for five consecutive days. Finally, 24 h after the last day of spatial learning training, a memory test was conducted. The escape platform was removed and the rats were released from the opposite point. They swam freely for 30 s. All of the trials were recorded and analyzed by Smart v3.0 video-tracking software (Panlab, Barcelona, Spain). Some of the recorded variables were latency to escape, speed, distance, and time spent in the different areas (quadrants, peripheria or thigmotaxis, and platform area). The values were expressed as the mean ± SEM.

### Measurement of Endogenous Hippocampal Aβ Levels

Hippocampi from control or DDVP-treated rats were weighed and homogenized in eight volumes of cold solution A, which consisted of 5 M guanidine HCl dissolved in 50 mM Tris-HCl, pH 8.0. This homogenate was maintained at room temperature for 3–4 h and then diluted with solution B, which consisted of 0.2 g/L KCl, 0.2 g/L KH_2_PO_4_, 8 g/L NaCl, 1.150 g/L HPO_4_, 5% BSA, and 0.03% Tween-20 at pH 7.4, supplemented with 1× protease inhibitor cocktail containing AEBSF, aprotinin, E64, EDTA, and leupeptin (Calbiochem, catalog number 539131). Solution B was added until reaching a final guanidine concentration of 0.1 M or lower. Then, the samples were centrifuged at 16000 ×*g* for 20 min at 4°C, and the supernatants were maintained on ice until Aβ quantification. For Aβ_1-40_, we used an ELISA kit from Invitrogen (catalog number KMB3481) that consists of a double ELISA antibody approach combined with colorimetric detection. In this kit, the wells are coated with a monoclonal antibody that recognizes the N-terminus of mouse Aβ. Samples and standards (provided in the kit) containing the Aβ antigen were added at the appropriate dilutions to the wells (100 μL in each well). The plate was incubated for 2 h at room temperature for the binding of the Aβ antigen to the immobilized (capture) antibody. Then, the solution was decanted and discarded from the wells and subsequently the wells were washed four times with the washing solution provided in the kit. After washing, 100 μL of a solution containing a rabbit antibody specific for the C-terminus of the Aβ_1-40_ sequence was added to the wells, except for the blanks. The plate was covered and incubated for 1 h at room temperature. After washing the wells four times, 100 μL of horseradish-peroxidase-labeled anti-rabbit antibody was added to each well except for the blanks. The plate was covered and incubated for 30 min at room temperature and then the wells were again washed four times. After washing, 100 μL of a solution containing the stabilized chromogen was added to each well and incubated for 20–30 min at room temperature in the dark. The reaction mixture turned blue. The reaction was stopped by adding 100 μL of stop solution to each well, at which point the reaction mixture turned yellow. Finally, the absorbance was recorded at 450 nm relative to the blanks that contained 100 μL of each stabilized chromogen solution and stop solution. The same procedure was performed for quantification of Aβ_1-42_, using the ELISA kit from Invitrogen (catalog number KMB3441).

### Statistical Analysis

All electrophysiological recordings of rat hippocampal slices were performed using slices from at least three different animals for each set of conditions (control and DDVP-exposed slices). The data were analyzed using the program InStat (GraphPad Software Inc., United States). A probability level of 0.05 or less was considered significant. For the Morris water maze experiments, ANOVA and Student’s *t*-test were used for the analysis. When the Shapiro–Wilk test showed a non-normal distribution of the data, the Mann–Whitney *U*-test was performed. Data were considered statistically significant when *p* ≤ 0.05.

## Results

### Long-Term Exposure to a Low Dose of DDVP (0.1 mg/kg) Enhances the LTP Measured at the *Stratum pyramidale* of the CA1 Area of the Rat Hippocampus

**FIGURE 1:**
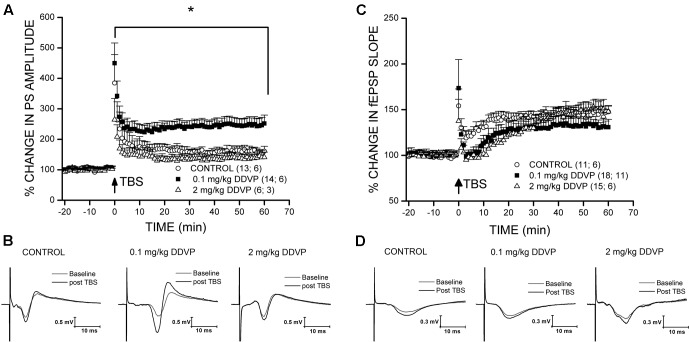
Effect of sub-chronic DDVP treatment on LTP elicited in the *stratum pyramidale* and *stratum radiatum* of the rat hippocampus. For each experiment, the percentage potentiation was calculated with respect to the mean of the baseline response. The number of slices and animals analyzed are indicated in parentheses besides each treatment condition. **(A)** LTP recordings in the *stratum pyramidale* of slices obtained from control animals (open circles), and animals treated with 0.1 mg/kg (black squares), or 2 mg/kg (open triangles) of DDVP. The animals treated with 0.1 mg/kg DDVP showed an enhanced LTP relative to the control animals (^∗^*p* < 0.05, unpaired *t*-test). **(B)** Representative traces of population spikes (PS) before (gray traces) and after TBS (black traces) for each condition. **(C,D)** Long-term potentiation recordings in the *stratum radiatum*. The symbols used are similar to those in **(A,B)**.

To test the effects of long-term *in vivo* exposure to various doses of DDVP on synaptic plasticity, we performed LTP experiments in hippocampal slices obtained from DDVP-treated rats and control animals injected with vehicle (corn oil) alone. Recordings of fEPSP and PS amplitudes were performed on the *stratum radiatum* and the *stratum pyramidale*, respectively, of the CA1 hippocampal area. **Table 1** shows the results of the LTP experiments performed on hippocampal slices obtained from control animals and those treated with a range of DDVP doses from 0.03 to 6 mg/kg. The values corresponding to the baseline (pre-TBS) and those obtained after the TBS (post-TBS) are shown and correspond to the mean of fEPSP or PS obtained during 10 min of data acquisition for pre-TBS and during 60 min of data acquisition for post-TBS. As can be seen, a significant LTP was achieved in recordings performed in the *stratum radiatum* and in the *stratum pyramidale* of slices obtained from control animals and from those exposed to all of the DDVP doses studied. However, only in the *stratum pyramidale* of CA1 did we find a significant enhancement of LTP in slices from animals treated with the 0.1 mg/kg dose, with respect to the control animals. In **Figure 1A**, a comparison between the effect on LTP of a low dose of DDVP (0.1 mg/kg) and a high dose (2 mg/kg) is presented relative to the control conditions. It can be seen that *in vivo* exposure to 0.1 mg/kg DDVP induced an enhanced LTP in the *stratum pyramidale* of 245.8 ± 21.7% (S.E.M., *n* = 14 slices, 6 rats, black squares) compared to that obtained in control rats (170.7 ± 14.8%, S.E.M., *n* = 13 slices, 6 rats, open circles, *p* < 0.05, unpaired *t*-test). On the contrary, as seen in **Figure 1C**, when the LTP was recorded in the *stratum radiatum*, the enhancement was not observed, with the LTP magnitude at 0.1 mg/kg DDVP being similar to that under control conditions (130.6 ± 6.7%, S.E.M., *n* = 18 slices, 11 rats, black squares, and 145.2 ± 3.3%, S.E.M., *n* = 11 slices, 6 rats, open circles, respectively; *p* = 0.15, unpaired *t*-test). Exposure to 2 mg/kg DDVP did not produce any enhancement in LTP at either of the recording sites, *stratum pyramidale* or *stratum radiatum* (compare **Figure 1A** with **Figure 1C**, open triangles).

**Table 1:** Effect of various doses of sub-chronic DDVP treatment on long-term potentiation recorded *ex vivo* in the *stratum radiatum* or *stratum pyramidale* of the CA1 area of rat hippocampal slices.

DDVP (mg/kg)	*n* (slices; animals)	Pre TBS	SEM pre TBS	Post-TBS	SEM post-TBS	*p*	ANOVA (vs. control)
***Stratum radiatum***
0	11;6	100.37	0.78	145.23	3.28	^∗∗∗^	
0.03	4;3	100.36	1.56	132.37	2.92	^∗∗^	ns
0.1	18;11	102.9	2.48	130.62	6.73	^∗∗^	ns
0.5	10;5	101.64	0.96	130.91	8.74	^∗^	ns
2	8;3	101.33	0.73	147.04	7.85	^∗∗^	ns
6	15;6	102	1.05	148.27	6.43	^∗∗^	ns
***Stratum pyramidale***
0	13;6	101.88	0.87	170.7	14.83	^∗∗^	
0.03	9;4	101.12	0.75	138.34	11.9	^∗^	ns
0.1	14;6	101.57	1.48	245.82	21.74	^∗∗^	^∗^
0.5	6;4	101.72	0.99	167.1	10.74	^∗∗∗^	ns
2	6;3	100.56	0.57	140.52	16.75	^∗^	ns
6	3;2	100.18	0.86	128.49	6.1	^∗^	ns

*The post-TBS values correspond to percentages relative to the normalized pre-TBS values. ^∗^*p* < 0.05, ^∗∗^*p* < 0.01, ^∗∗∗^*p* < 0.001. Paired *t*-test, except in ANOVA analysis*.

### Long-Term Exposure to a Low Dose of DDVP (0.1 mg/kg) Inhibits APEH and Not AChE Activity Measured in Homogenates of the Rat Hippocampus

**FIGURE 2:**
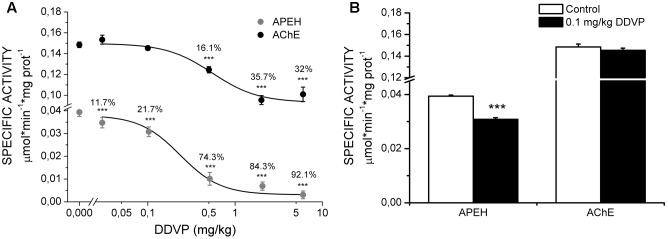
Quantification of specific activities of APEH and AChE. **(A)** Dose–response curves for the inhibition of hippocampal specific activities of APEH (gray circles) and AChE (black circles) upon administration of various doses of DDVP to different groups of animals. Significant percentages of inhibition are showed above each activity point. The dose–response curves of inhibition were adjusted to a Hill equation (the IC_50_ values for APEH and AChE were 0.234 ± 0.092 mg/kg and 0.546 ± 0.146 mg/kg, respectively. **(B)** Specific activities for the two enzymes under control conditions and at a DDVP dose of 0.1 mg/kg. Only APEH was significantly inhibited at this dose. ^∗∗∗^*p* < 0.001, unpaired *t*-test.

The effect of DDVP exposure on LTP was correlated with the levels of APEH and AChE enzymatic activities. For this, we used the homogenates of hippocampal slices from the control and DDVP-treated rats recovered from the recording chamber. **Figure 2A** shows the inhibitory effect of various doses of DDVP on brain APEH (gray circles) and AChE (black circles) activities. It can be seen that AChE seemed to reach a steady state of inhibition at 2 mg/kg DDVP. At this dose, an inhibition of 35.7 ± 4.3% was obtained, which did not change significantly at the highest dose of DDVP tested (6 mg/kg, 32 ± 7% inhibition, *p* = 0.52). On the contrary, the brain APEH activity was almost totally inhibited in rats exposed to 6 mg/kg DDVP (92.1 ± 1.7% inhibition). The specificity of the inhibitory effect of DDVP toward APEH and not AChE activity was obtained at the dose of 0.1 mg/kg. At this dose, brain APEH activity displayed a 21.7 ± 0.61% inhibition (*p* < 0.001), whereas AChE activity remained unaffected (*p* = 0.13) (**Figure 2B**). Finally, the profile of the inhibitory effect of DDVP over both enzymatic activities was adjusted to fit a Hill equation, yielding IC_50_ values of 0.234 ± 0.092 mg/kg and 0.546 ± 0.146 mg/kg for APEH and AChE activities, respectively.

### Long-Term Exposure to a Low-Dose of DDVP (0.1 mg/kg) Improves the Spatial Learning and Memory in the Water Maze Test

**FIGURE 3:**
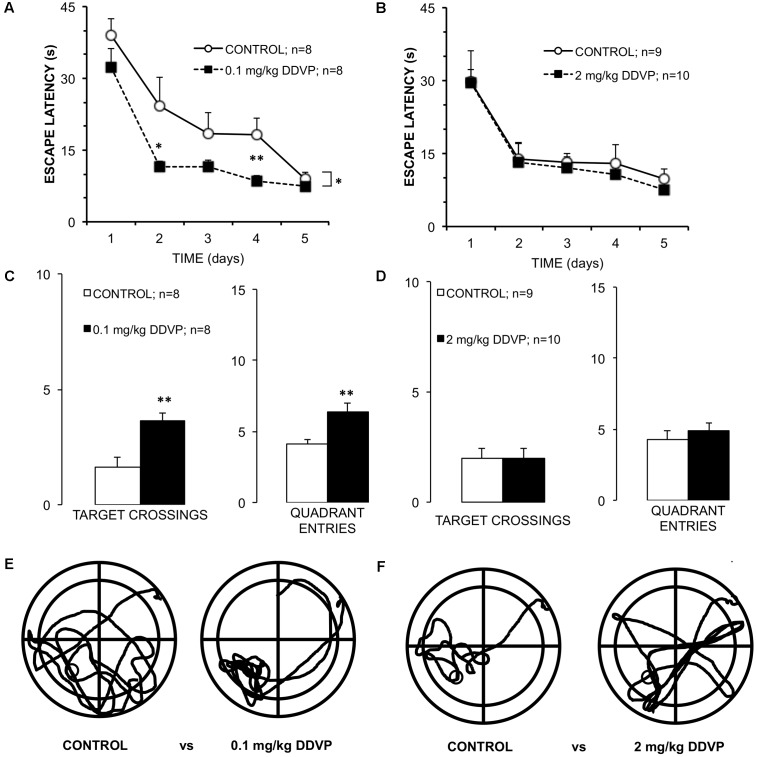
Differential effects of low and high DDVP doses on learning and memory. **(A,B)** Graphs of the escape latency (time to reach the submerged platform) for groups of rats treated with **(A)** 0.1 mg/kg DDVP (black squares), **(B)** 2 mg/kg DDVP (black squares), and their control groups (open circles). Rats treated with the low DDVP dose showed an improved escape latency starting from the 2nd day of training. **(C,D)** Results of the memory tests performed 24 h after the learning test. **(C)** Rats treated with 0.1 mg/kg DDVP (black columns) showed a significantly higher number of target crossings and quadrant entries compared to **(D)** rats treated with 2.0 mg/kg DDVP (black columns), which did not show significant differences with respect to the controls. **(E,F)** Representative trajectories of rats swimming for each set of conditions. Rats treated with 0.1 mg/kg DDVP showed improved escape latency and quickly found the platform location. ^∗^*p* < 0.05, ^∗∗^*p* < 0.01. See the text in Results section for details.

To evaluate whether the enhancement of LTP induced by low doses of DDVP in the *stratum pyramidale* was correlated with an improvement in learning and memory, behavioral experiments were conducted using the Morris water maze test. As such, three additional groups of rats were administered with daily injections of corn oil (control group) or DDVP at 0.1 mg/kg (low-dose group) or 2 mg/kg (high-dose group) for 28 days, resembling the same schedule as the rats subjected to the electrophysiological experiments. **Figure 3A** shows the time taken to reach the platform (latency to escape) of the control and low-dose animals (0.1 mg/kg) over the 5 days of the spatial learning task. A 2 × 5 (doses × training days) ANOVA showed a between-group effect (*p* = 0.02) and a repeated-measure effect of the training days (*p* < 0.001). The low-dose group showed a lower latency in finding the platform through the 5 days of training compared to the control animals, even though significant reductions in the time taken to reach the platform were observed for both groups respect to the 1st day of the spatial learning task. These results proved a good spatial learning for both groups, although the low-dose group exhibited a faster learning than the control group. Analyses of each training day showed a significant difference between the DDVP and control groups at day 4 (*p* = 0.01; Mann–Whitney *U*-test). This result is consistent with the data obtained in the memory test (without platform) carried out 24 h after the last training day. As shown in **Figure 3C**, the number of crossings in the platform area and the number of entries in the quadrant where the platform was located were monitored. Rats belonging to the low-dose group showed an increased number of crossings in the platform area (*p* = 0.002, *t*-test) and an increased number of entries to the target quadrant (*p* = 0.007, *t*-test) compared to the control group. Additional measurements of spatial memory are shown in **Supplementary Figure S1**. Finally, **Figure 3E** shows typical trajectories for rats from each group during the memory test, demonstrating the greater accuracy in locating the platform of the rat injected with the low dose. In conclusion, rats injected with low doses of DDVP (0.1 mg/kg) exhibited faster learning and better spatial memory in the Morris water maze than control rats. We discard the possibility of these learning and memory improvements being attributed to differences in visual, motor, or motivational skills between the groups, because the analysis of the latency to reach the platform in the visual cue task did not show any difference between the groups (*p* = 0.36, *t*-test). Furthermore, there were no differences in the mean swimming speed (*p* = 0.89, 2 × 5 ANOVA), nor in the time spent in the periphery or thigmotaxis (*p* = 0.08, 2 × 5 ANOVA) during the spatial learning task and the memory test.

In contrast to the above results, **Figure 3B** shows the latency to reach the platform for the high dose (2 mg/kg DDVP) and control groups during the spatial learning task. A 2 × 5 (doses × training days) ANOVA showed only a repeated-measures effect for the training days (*p* < 0.001). In the same way, the memory test showed no difference between groups (**Figure 3D**). **Figure 3F** shows representative examples of the trajectories of a rat injected with the high DDVP dose and a control rat during the memory test. In conclusion, there were no differences between the groups in terms of the latency to reach the platform, the visual cue task, the swimming speed, or in the other parameters assessed during the memory test. This supports the idea that DDVP, at these doses, is not affecting visual, motor, or motivational variables, and we can therefore discard a bias in the normal learning during the Morris water maze test.

### Long-Term Exposure to a Low Dose of DDVP (0.1 mg/kg) Does Not Affect Synaptic Excitability in the CA1 Area of the Rat Hippocampus and Does Not Induce Changes at the Presynaptic Level

**FIGURE 4:**
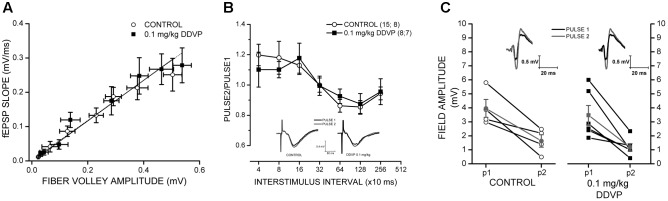
Effect of 0.1 mg/kg DDVP on synaptic plasticity parameters. **(A)** Effect of DDVP on synaptic efficacy by the measurement of input/output curves obtained from hippocampal tissues of control rats (open circles) and those treated with 0.1 mg/kg DDVP (black squares). The treatment with DDVP did not produce any change in the synaptic efficacy of the Schaffer collateral → CA1 pathway. **(B)** Interstimulus interval (ISI) curves for hippocampal slices obtained from control (black squares) and DDVP-treated animals (open circles). Paired-pulse facilitation was observed for both conditions at interstimulus intervals below 160 ms. The insets show representative traces of fEPSPs for the control and DDVP-treated animals obtained at a interstimulus interval of 320 ms. Black traces indicate the first pulse (P1) and gray traces indicate the second pulse (P2). **(C)** Paired-pulse inhibition (PPI) experiments for control conditions (open circles) and DDVP treatment (black squares). The interstimulus interval for these experiments was 13 ms. The PS amplitudes obtained for the first and second pulses are shown. For both conditions, the means of P1 and P2 (gray circles and gray squares for control and DDVP treatment, respectively) are shown. The slopes of the lines connecting the means of P1 and P2 for the two conditions do not show statistical differences. The insets show representative PS traces. Black traces indicate the first pulse (P1) and gray traces indicate the second pulse (P2).

To determine if the exposure to DDVP affects the synaptic efficacy of the Schaffer collateral → CA1 pathway, input/output ratios were measured in response to single electrical stimulation, and the control and DDVP-treated groups were compared. Three fEPSP responses were collected and averaged for each stimulus increment. To construct input/output curves, the amplitude of the fiber volley and the slope of the fEPSP were averaged across all slices measured for each group (control and 0.1 mg/kg DDVP). No significant differences were found between the control and low-dose-treated rats (**Figure 4A**), suggesting that these two groups had similar synaptic densities at the *stratum radiatum* of CA1 and displayed equivalent excitability in response to single electrical stimulation (*y* = 0.51*x* + 0.01 for the control and *y* = 0.54*x* + 0.01 for slices from the DDVP-treated rats, *p* = 0.74).

To analyze the presynaptic function and GABAergic interneuron activity, we performed PPF and PPI experiments (**Figures 4B,C**, respectively). In slices from the DDVP-treated rats, the paired-pulse ratio obtained in PPF experiments at a broad range of interstimulus intervals did not differ significantly from slices from the control rats (**Figure 4B**); for example, for paired pulses applied 40 ms apart, no significant difference in the ratios was observed for the control and DDVP-treated rats (1.2 ± 0.07 versus 1.1 ± 0.12, respectively, *p* = 0.84, **Figure 4B**). The same was observed in the PPI experiments, where no difference was found between the two conditions. In **Figure 4C**, it can be seen that the ratio of the paired pulses applied 13 ms apart did not differ significantly between the two groups (0.43 ± 0.11 versus 0.38 ± 0.08, respectively, *p* = 0.73, **Figure 4C**). For further details about the rationale of PPF and PPI experiments, see Olmos et al. (2009).

### DDVP Exposure Causes an Increase in the Endogenous Concentration of Aβ_1-40_ in the Hippocampus of Treated Rats

**FIGURE 5:**
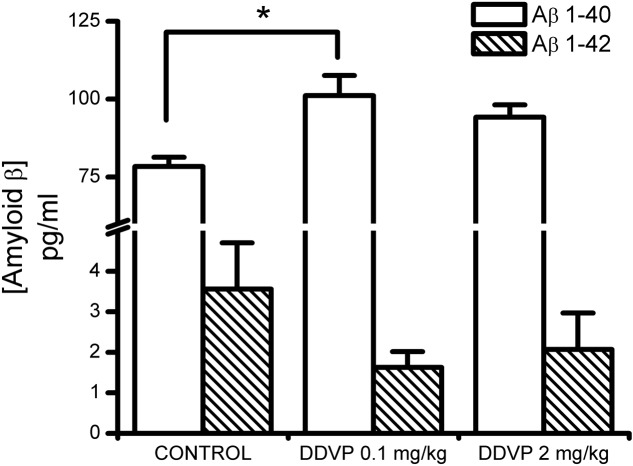
Effect of sub-chronic treatments with low and high DDVP doses on the endogenous concentrations of Ab_1-40_ and Ab_1-42_. Both DDVP doses produced increases in the endogenous concentration of Aβ_1-40_, which were statistically significant for the low-dose condition. ^∗^*p* < 0.05.

Finally, with the aim of determining whether the effects described above are related to changes in the endogenous concentrations of Aβ_1-40_ or Aβ_1-42_, we measured the concentrations of these two peptides in homogenates obtained from hippocampi of control and DDVP-treated rats. As shown in **Figure 5**, it can be seen that administration of 0.1 or 2 mg/kg DDVP caused an increase in the endogenous concentration of Aβ_1-40_ (*F* = 5.17, *p* = 0.022), being statistically significant only the increase observed with 0.1 mg/kg DDVP (*p* = 0.027, Bonferroni *post hoc* test). Endogenous concentration of Aβ_1-42_ remained unaffected (*F* = 1.36, *p* = 0.304).

## Discussion

Despite the fact that much time has passed since it was discovered that Aβ peptides are produced by neurons under normal metabolic conditions (Haass et al., 1992; Shoji et al., 1992), these molecules have traditionally been considered to have a negative role on synaptic transmission and plasticity, inducing disruption of these processes and being associated with the development of neurodegenerative diseases such as Alzheimer’s disease [for a review about this topic, see Puzzo et al. (2015)]. In this study, we have demonstrated that in young, healthy brains, an increase of endogenous Aβ_1-40_ concentrations has positive effects on synaptic plasticity and memory, this effect being correlated with a slight but significant inhibition of APEH activity. The results reported here are the continuation of a previous work, in which we demonstrated that acute inhibition of APEH activity by DDVP can enhance LTP at a specific dose and time of exposure (Olmos et al., 2009). The major contribution of the present study was to demonstrate that the same effects can be accomplished *in vivo* after sub-chronic treatment of young rats (1–2 months) with a low dose of DDVP (0.1 mg/kg). Using this protocol, we were able to obtain an improvement in LTP and spatial memory in which only APEH activity was significantly inhibited (∼20%) while AChE activity remained unaffected. The results reported here explain the observations made by Van der Staay et al. (1996), in which improvements in spatial learning were achieved at 0.03 mg/kg DDVP and AChE inhibition was observed only from 1 mg/kg administered p.o. to young adult male Wistar rats. Therefore, taking in consideration our results, the improvements in spatial learning observed by Van der Staay et al. (1996) can now be attributed to APEH inhibition and not to AChE inhibition. Our results are also consistent with those of Yamin et al. (2007, 2009), in which APEH found in the conditioned medium of the human neuroblastoma cell line can degrade monomers, dimers, and trimers of Aβ_1-40_ *in vitro*. The increase in endogenous Aβ_1-40_ concentrations that we observed after 28 days of DDVP treatment coincides with the inhibition of APEH in the same tissue. Finally, our results are also partially consistent with those reported by Liu et al. (2002), who described an increase in the concentrations of both Aβ_1-40_ and Aβ_1-42_ in the hippocampi of transgenic mice after treatment with metrifonate, the pro-drug of DDVP, with the increase of Aβ_1-40_ being greater than that of Aβ_1-42_. Contrary to this report, our results indicate that in young, normal rat brains the increase in concentration is only observed for Aβ_1-40_ and not for Aβ_1-42_. Furthermore, an increase in Aβ_1-40_ concentration was also observed for the higher DDVP dose tested (2 mg/kg), although this was not statistically significant. It is important to keep in mind that with the dose of 2 mg/kg we observed the inhibition of both enzymes, APEH and AChE. This is very interesting and highlights the necessity of both mechanisms – increasing the concentration of Aβ_1-40_ and specific inhibition of APEH and not of AChE – to attain improvements in synaptic plasticity and spatial memory.

It is important to mention that if Aβ peptides are one of the endogenous substrates of APEH, their degradation could not be explained by the exopeptidase activity of APEH toward *N*-acylated peptides but by its endopeptidase activity. The endopeptidase activity of APEH has been observed in the enzyme from erythrocytes (Fujino et al., 2000a) and in the truncated 55 kDa enzyme found in the lens (Senthilkumar et al., 2001). The endopeptidase activity of APEH has not been described in brain tissue, although the enzyme secreted by the human neuroblastoma cell line cleaves Aβ_1-40_ at the amino acid residues 13, 14, and 19 (Yamin et al., 2009). The biochemical aspects that modulate the type of catalytic activity of APEH in the brain (i.e., exo- or endopeptidase) are interesting issues for further investigation.

The positive effect of Aβ peptides on synaptic plasticity has been well established by a handful of studies. It has been reported that exogenously added picomolar amounts of Aβ_1-40_ led to a neurotrophic effect in cell cultures (Yankner et al., 1990) and the same amounts of Aβ_1-42_ had a positive effect on synaptic plasticity (Puzzo et al., 2008). Later, using a strategy of depletion of endogenously produced Aβ peptides and subsequent recovery by adding Aβ_1-42_, the latter authors demonstrated that this peptide is necessary for synaptic plasticity in the mice hippocampus (Puzzo et al., 2011). Interestingly, the mechanism responsible for the effects of Aβ on synaptic plasticity would be mediated by α_7_ nicotinic acetylcholine receptors (β_7_nAChRs), as it is known that Aβ peptides modulate the activity of this receptor (Parri et al., 2011; Puzzo and Arancio, 2013). Interestingly, we have observed previously that α_7_nAChRs are involved in the boost of LTP observed after acute exposure to DDVP (Olmos et al., 2009). α_7_nAChRs are homopentameric ion channels that are highly permeable to Ca^2+^ (Séguéla et al., 1993; McGehee, 1999) and widely distributed in the brain (Karlin, 2002; Wallace and Porter, 2011), where they play an important role in normal and pathogenic cognitive processes (Alkondon and Albuquerque, 2004; Lewis et al., 2017). Functional and ultrastructural studies of the CA1 region of the hippocampus have demonstrated that α_7_nAChRs are located in both presynaptic and post-synaptic sites (Fabian-Fine et al., 2001), especially in GABAergic interneurons (Wanaverbecq et al., 2007). Moreover, these receptors have been found in post-synaptic densities in excitatory synapses of the somatosensory cortex (Levy and Aoki, 2002). The final effect mediated by α_7_nAChRs on synaptic plasticity and behavioral tests depends on the Aβ concentration, showing the modulation of α_7_nAChRs by Aβ peptide possesses a biphasic profile, in which picomolar concentrations have a boosting effect on functional parameters whereas nanomolar concentrations have the opposite effect (Parri et al., 2011). This kind of effect has been observed for both the Aβ_1-40_ and Aβ_1-42_ peptides. Recently, Lawrence et al. (2014) reported that Aβ_1-15_, another fragment of amyloid precursor protein (APP) processing, can boost synaptic plasticity and behavior through the modulation of α_7_nAChRs. Taking in consideration these antecedents, it is necessary to establish the involvement of a7nAChRs in the mechanism responsible for the enhancement of synaptic plasticity and memory caused by APEH inhibition *in vivo*.

For understanding the physiological role of Aβ peptides in synaptic transmission and the relevance for controlling the homeostasis of its endogenous concentrations, it is important to determine the putative role of APEH in this process and if the enzyme co-localizes with Aβ. In a previous report from our group using subcellular fractionation approaches, we demonstrated that APEH can be found in synapses isolated from the rat telencephalon and is mainly associated to presynaptic components (Sandoval et al., 2012). Furthermore, it has also been reported that APEH can be found in the serum-free conditioned medium of the neuroblastoma cell line, thus indicating that the enzyme can be secreted (Yamin et al., 2007). Interestingly, it has been shown that Aβ production occurs in late and early endosomes once APP has been endocytosed and Aβ peptides produced inside cells are then released to the extracellular parenchyma in a process dependent on synaptic activity (Cirrito et al., 2005, 2008). Determination of the co-localization of APEH with APP in the endocytic compartment is an interesting issue to be addressed in future studies.

It is important to note that the LTP enhancement was only obtained in the *stratum pyramidale* and not in the *stratum radiatum* (**Figure 1** and **Table 1**). In rat hippocampal slices, the glutamatergic synapse is produced between the axon terminals projected from pyramidal neurons located at the CA3 area to the dendrites of pyramidal neurons of the CA1 area. This pathway is called the Schaffer collateral pathway and the point of synaptic contact is the *stratum radiatum*. The *stratum pyramidale* corresponds to a layer constituted by the soma of pyramidal neurons. This difference in the effect of DDVP depends on the layer in which field potentials are recorded and could possibly be due to a diminished inhibitory input of GABAergic interneurons to the soma of CA1 pyramidal neurons. However, our results did not indicate any effect of DDVP treatment on GABAergic interneurons, as was evidenced in the extracellular PPI experiments (**Figure 4**). Another possibility relating to this is the reduction in other shunting conductances that could explain the significantly enhanced field PS responses measured in the *stratum pyramidale* compared to fEPSP measured in the *stratum radiatum*. For example, it is known that the activation of metabotropic glutamate receptors (mGluRs) present in hippocampal excitatory synapses can activate a signal transduction pathway that results in a reduction of potassium conductances (Charpak and Gähwiler, 1991).

This report has presented for the first time evidence that supports the role of APEH in the control of the homeostasis of endogenous brain Aβ concentrations and its effects on synaptic plasticity and behavior. Our results also demonstrate that APEH is selectively inhibited at low doses of DDVP, making this enzyme rather than AChE the primary pharmacological target of DDVP. In conclusion, these results indicate that APEH is an interesting pharmacological target for cognitive enhancement.

## Author Contributions

All the authors have contributed significantly to the submitted work. RS, CR, BM, UW, and FP designed the experiments. GG-R, FG, EA, and DR-E performed experiments and analyzed data. FP wrote the manuscript. UW and BM made critical revision of the manuscript.

## Conflict of Interest Statement

The authors declare that the research was conducted in the absence of any commercial or financial relationships that could be construed as a potential conflict of interest.

## Supplementary Material

FIGURE S1 Differential effects of low and high DDVP doses on spatial memory measurements **(A–D)** Effect of treatment with 0.1 mg/kg DDVP (black columns) on latency to escape **(A)**, distance to platform **(B)**, time in platform **(C)**, and distance in platform **(D)**. As it can be observed, treated rats spent more time in platform respect to control group **(C, D)**. **(E–H)** Effect of treatment with 2.0 mg/kg DDVP (black columns) on latency to escape **(E)**, distance to platform **(F)**, time in platform **(G)**, and distance in platform **(H)**. Only the parameter “distance to platform” resulted to be significantly decreased in DDVP treated rats respect to control group **(F)**. ^∗^*p* < 0.05, unpaired *t*-test.
