# Supplementary figures and images for: In Vivo Sub-chronic Treatment with Dichlorvos in Young Rats Promotes Synaptic Plasticity and Learning by a Mechanism that Involves Acylpeptide Hydrolase Instead of Acetylcholinesterase Inhibition. Correlation with Endogenous β-Amyloid Levels
Source: Front Pharmacol. 2017 Jul 25;8:483. doi: 10.3389/fphar.2017.00483 (PMC5524899; doi:10.3389/fphar.2017.00483)

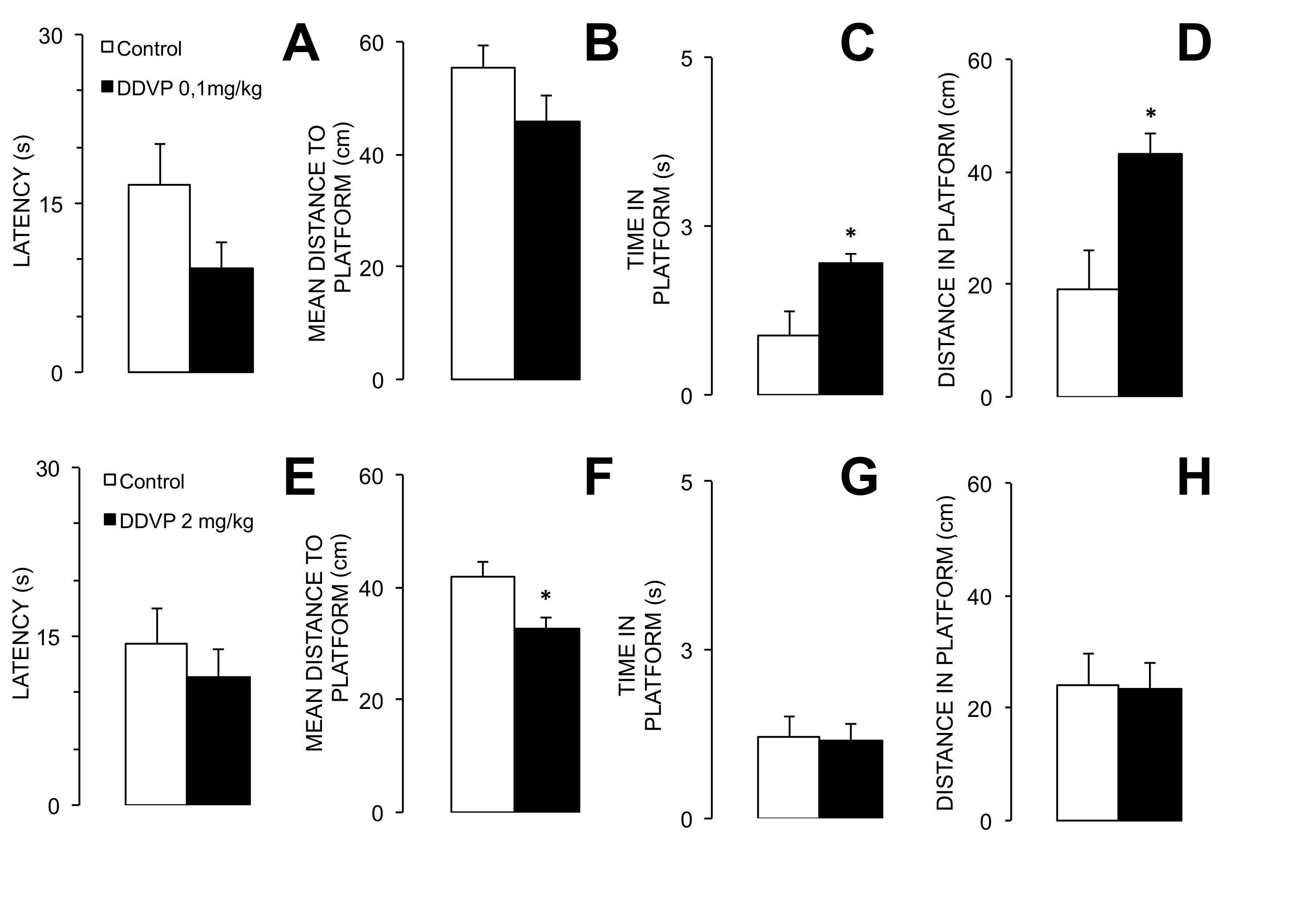

Supplement: FIGURE S1 — Differential effects of low and high DDVP doses on spatial memory measurements (A–D) Effect of treatment with 0.1 mg/kg DDVP (black columns) on latency to escape (A), distance to platform (B), time in platform (C), and distance in platform (D). As it can be observed, treated rats spent more time in platform respect to control group (C, D). (E–H) Effect of treatment with 2.0 mg/kg DDVP (black columns) on latency to escape (E), distance to platform (F), time in platform (G), and distance in platform (H). Only the parameter “distance to platform” resulted to be significantly decreased in DDVP treated rats respect to control group (F). ∗p < 0.05, unpaired t-test. [file Image_1.JPEG]
